# A Mathematical Model of the Mouse Ventricular Myocyte Contraction

**DOI:** 10.1371/journal.pone.0063141

**Published:** 2013-05-09

**Authors:** Paula D. Mullins, Vladimir E. Bondarenko

**Affiliations:** 1 Department of Mathematics and Statistics, Georgia State University, Atlanta, Georgia, United States of America; 2 Neuroscience Institute, Georgia State University, Atlanta, Georgia, United States of America; Temple University, United States of America

## Abstract

Mathematical models of cardiac function at the cellular level include three major components, such as electrical activity, Ca^2+^ dynamics, and cellular shortening. We developed a model for mouse ventricular myocyte contraction which is based on our previously published comprehensive models of action potential and Ca^2+^ handling mechanisms. The model was verified with extensive experimental data on mouse myocyte contraction at room temperature. In the model, we implemented variable sarcomere length and indirect modulation of the tropomyosin transition rates by Ca^2+^ and troponin. The resulting model described well steady-state force-calcium relationships, dependence of the contraction force on the sarcomere length, time course of the contraction force and myocyte shortening, frequency dependence of the contraction force and cellular contraction, and experimentally measured derivatives of the myocyte length variation. We emphasized the importance of the inclusion of variable sarcomere length into a model for ventricular myocyte contraction. Differences in contraction force and cell shortening for epicardial and endocardial ventricular myocytes were investigated. Model applicability for the experimental studies and model limitations were discussed.

## Introduction

Cardiac cell functions include the interaction of several major subsystems, including those responsible for the generation of electrical activity, Ca^2+^ dynamics, and cardiac contraction. Experimental data from diseased hearts or obtained at fast pacing rates show that the changes in one of the subsystems can lead to abnormal behavior in others. For example, dysfunction of the L-type Ca^2+^ channel, as in Timothy syndrome when the channel’s inactivation is significantly reduced, affects Ca^2+^ handling in cardiac cells [1], [2] resulting in cardiac arrhythmias. Heterogeneities in cellular electrical activities in the heart, dysfunction of K^+^ channels, or acidosis can also produce pro-arrhythmic behavior, such as action potential propagation block, re-entry, Ca^2+^ alternans, and irregular contractions [3], [4]. In particular, instability of Ca^2+^ dynamics (alternans) can lead to the action potential alternans [5] and alternans in mechanical contraction [6]. Therefore, understanding interactions of the major cardiac cell subsystems and mechanisms of their pro-arrhythmic activity is of great importance.

Mathematical modeling of electrical activity, Ca^2+^ dynamics, and cardiac contraction is a supplementary tool for experimentalists in order to understand mechanisms of pro-arrhythmic activity in the heart. There are several models for cardiac myocyte contraction that have been developed for different species. Such models were developed for guinea pig [7], [8], rabbit [9], canine [10], and mouse [11] ventricular myocytes. The models include experimentally-verified sets of ionic currents, Ca^2+^ dynamics, and contractile parameters for cardiac cells of the particular species.

**Figure 1:**
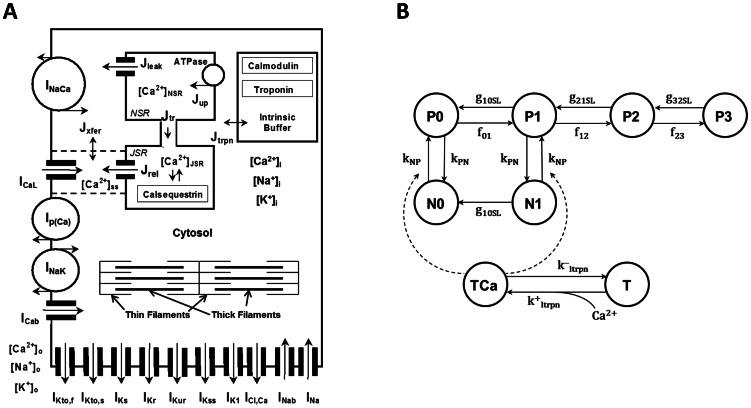
Schematic diagram of the mouse model cell and Markov model for force generation. (A) Mouse model ionic currents and Ca^2+^ fluxes as presented by Bondarenko et al. [19]. Transmembrane currents are the fast Na^+^ current (I_Na_), the L-type Ca^2+^ current (I_CaL_), the sarcolemmal Ca^2+^ pump (I_p(Ca)_), the Na^+^/Ca^2+^ exchanger (I_NaCa_), the rapidly recovering transient outward K^+^ current (I_Kto,f_), the slowly recovering transient outward K^+^ current (I_Kto,s_), the rapid delayed rectifier K^+^ current (I_Kr_), the ultrarapidly activating delayed rectifier K^+^ current (I_Kur_), the noninactivating steady-state voltage activated K^+^ current (I_Kss_), the time-independent K^+^ current (I_K1_), the slow delayed rectifier K^+^ current (I_Ks_), the Na^+^/K^+^ pump (I_NaK_), the Ca^2+^-activated chloride current (I_Cl,Ca_), the Ca^2+^ and Na^+^ background currents (I_Cab_ and I_Nab_). I_stim_ is the external stimulation current. The Ca^2+^ fluxes within the cell are uptake of Ca^2+^ from the cytosol to the network sarcoplasmic reticulum (SR) (J_up_), Ca^2+^ release from the junctional SR (J_rel_), Ca^2+^ flux from the network SR (NSR) to junctional SR (JSR) (J_tr_), Ca^2+^ leak from the SR to the cytosol (J_leak_), Ca^2+^ flux from the subspace volume to the bulk myoplasm (J_xfer_), Ca^2+^ flux to troponin (J_trpn_). The model includes Ca^2+^ buffering by troponin and calmodulin in the cytosol and by calsequestrin in the SR. [Ca^2+^]_i_, [Na^+^]_i_, and [K^+^]i are the intracellular Ca^2+^, Na^+^, and K^+^ concentrations in cytosol; [Ca^2+^]_o_, [Na^+^]_o_, and [K^+^]_o_ are the extracellular Ca^2+^, Na^+^, and K^+^ concentrations. Contraction force F_contr_ develops due to interaction of thin and thick filaments in the cytosol. Thick filaments are composed of myosin, thin filaments consist of actin, tropomyosin, and interact with troponin. (B) State diagram of the Markov model for the force generation in mouse cardiac myofilaments [15]. Top states describe cross-bridge formation, bottom states describe Ca^2+^ binding to troponin. P0, P1, P2, and P3 are the permissive states; N0 and N1 are the nonpermissive states. TCa is Ca^2+^ bound troponin; T is unbound troponin.

Myocyte contraction is a complex process which involves activation of ionic currents, including L-type Ca^2+^ current, through which Ca^2+^ enters the cell and causes Ca^2+^ release from the intracellular Ca^2+^ store, the sarcoplasmic reticulum [12]. High intracellular Ca^2+^ concentration leads to an increase in Ca^2+^ bound by intracellular proteins (troponin, calmodulin) and changes the myofilament configuration, resulting in force development. Force generation involves conformational changes in thick (myosin) and thin (actin, tropomyosin, and troponin) filaments (Fig. 1A) resulting in an increase in their overlap. Myosin represents a polypeptide chain with globular heads, which constitute crossbridges that interact with thin filaments. Thin filaments are composed of long tropomyosin polypeptide chains, on which globular actin molecules aggregate in double-stranded helix with crossbridge binding sites. In a non-active configuration, troponin blocks crossbridge binding sites. Upon Ca^2+^ binding to troponin, troponin-tropomyosin complex exposes crossbridge binding sites which interact with myosin globular heads, thereby creating weak bonds. ATP molecules bound to actin release a phosphate group and transform weak bonds into strong bonds. This transformation results in a change of crossbridge conformation to a bent position and forces thick filaments to slide relative to thin filaments.

Because of the complexity of the contraction mechanism, most mathematical models use a significantly simplified description of this process [13]. They explore the Huxley two-state crossbridge model [14], extend it to a larger number of crossbridge states, and include direct and indirect interaction with troponin and variable sarcomere lengths [13], [15]. Such simplified description, for example, does not involve energy metabolism and interaction with mitochondria. The crossbridge models are further incorporated into cellular models, which include electrical activity, comprehensive Ca^2+^ dynamics [7], [9], [11], and energy metabolism [16], [17].

A mathematical model which describes mouse ventricular cell contraction for body temperature (310°K, or +37°C) was developed and published recently by Land et al. [11]. The model adjusted the cellular contraction model of Rice et al. [9] for mice and is primarily devoted to simulation of the whole heart contraction. While some experimental data is recapitulated by the model, such as time course of the myocyte contraction, the deviation of the simulated from the experimental data and model limitations are also discussed by the authors [11]. For example, absolute values of the simulated cellular contraction force are considerably larger than the measured contraction force for both physiological and room temperatures [18]. In addition, Land et al. [11] did not study contraction force-frequency relationships at the cellular level.

In this paper, we developed a new electromechanical model for mouse ventricular myocyte contraction at room temperature (298°K, or +25°C). We employed our previously published models for action potential and Ca^2+^ dynamics in mouse ventricular myocytes [19], [20], [21], [22], which were also developed for room temperature (298°K, or +25°C), and incorporated a myocyte contraction model from Rice et al. [15]. These models were successfully employed for simulations of proarrhythmic activities in mouse cardiac cells and tissues [21], [22]. In addition, in the Rice et al. [15] model, we implemented sarcomere length variation during twitch. We also explored the effects of heterogeneity of the electrical activity and Ca^2+^ dynamics in epicardial and endocardial cells on the contraction force generation and cell shortening. The resulting model was adjusted to fit experimental data on mouse ventricular cell contraction. Our model successfully reproduced steady-state force-calcium relationships for different sarcomere lengths; time courses of the Ca^2+^ transients, developed force, and cellular shortening; peak force-frequency and cell shortening-frequency relationships; and time-to-peak force and time-to-50% force relaxation. We also investigated and emphasized the importance of using variable sarcomere lengths in models of myocyte contraction. In the simulations, we compared both the absolute value of the contraction force and cellular shortening, and their normalized dependences to fit existing experimental data.

## Methods

A mathematical model for mouse ventricular myocyte contraction is a natural extension of our previously published models for action potential and Ca^2+^ dynamics in mouse ventricular myocytes [19], with model improvements from [20], [21], [22] (Fig. 1A), developed for room temperature (298°K, or +25°C). In this paper, we explore mouse ventricular myocyte models from the epicardial and endocardial regions of the heart [22]. Endocardial cells have more prolonged action potentials and larger intracellular [Ca^2+^]_i_ transients compared to epicardial cells [22]. We incorporated the Rice et al. [15] contraction model 4 in our model of electrical activity and Ca^2+^ handling [19], [20], [21], [22] (See Appendix S1) and adjusted model parameters to fit experimental data on myocyte contraction obtained for room temperatures.

The Rice et al. [15] model links Ca^2+^ dynamics and myocyte contraction (Fig. 1B). The model contains two nonpermissive tropomyosin states (N0 and N1) and four permissive tropomyosin states (P0, P1, P2, and P3). N0, N1, P0, P1, P2, and P3 are functions of time that describe probabilities of finding the model in that particular state. N0 is the rest state of the model, with no strongly bound crossbridges. When Ca^2+^ binds to the tropomyosin, it changes its conformation to a permissive state without strongly bound crossbridges (P0), which allows for strong binding of one (P1), two (P2), or three (P3) crossbridges. The model also includes one nonpermissive state with one strongly bound crossbridge even without a bound Ca^2+^ ion. All transition rates in the model are Ca^2+^-independent, except for k_NP_, which depends on the concentration of troponin with Ca^2+^ bound to a low-affinity binding site. Detailed analysis of several contraction models and the plausibility of different cooperative mechanisms was performed in [15]. The model which we adopted for the mouse ventricular myocyte contraction (Model 4 from [15]) gave the best fit to the existing experimental data for mice. The contraction model parameters for epicardial and endocardial cells are presented in the Supporting Information (Appendix S1).

Contraction force F_contr_ (in mN/mm^2^) was calculated using the equation [15]:
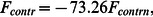
(1)where



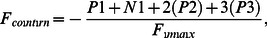
(2)

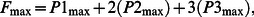
(3)

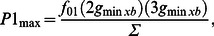
(4)

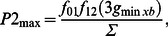
(5)

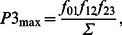
(6)

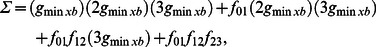
(7)

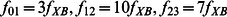
(8)


In equation (1), F_contrn_ is the normalized contraction force, and the coefficient −73.26 is obtained from fitting absolute values of the steady-state and dynamic experimental forces. For simulation steady-state force-calcium relationships (F-Ca), we used fixed values of the sarcomere lengths (SL), so that d(SL)/dt = 0, and changed intracellular Ca^2+^ concentration. We simulated F-Ca relationships for sarcomere lengths 1.9, 2.1, and 2.3 µm. In this case, F_contrn_ has time-independent magnitude.

For simulation twitch contraction, where F_contrn_ is time-dependent, we used Hooke’s law, the linear relationship between contraction/relaxation force and cell shortening/extension:
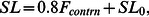
(9)where SL_0_ is the initial value of SL. In this case, sarcomere length becomes a function of time. To compare simulation data with experiments for cellular contraction, we estimated the variable cell length by
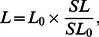
(10)where initial cell length L0 = 100 µm. For all simulations, we used extracellular Ca2+ concentration [Ca2+]o = 2 mM.

The electromechanical cardiac cell models were stimulated with different frequencies using a stimulus current (I_stim_ = 80 pA/pF, τ_stim_ = 0.5 ms) for at least 200,000 ms to reach a quasi-steady state. Simulated data of intracellular [Ca^2+^]_i_ transients, myocyte contraction force F_contr_, and sarcomere length SL on the interval from 192,000 to 200,000 ms were compared to extensive experimental data.

The model consists of 51 ordinary differential equations (see Appendix S1). Differential equations are solved by fourth-order Runge-Kutta method with time step 0.0001 ms. The model was implemented as an original Intel FORTRAN 90 code, which was run under SUSE Linux on a Dell Precision Workstation T3500 (Intel Xeon Processor W3670, 3.20 GHz, 8 GB RAM).

## Results

### Steady-state Force-calcium Relationships

**Figure 2:**
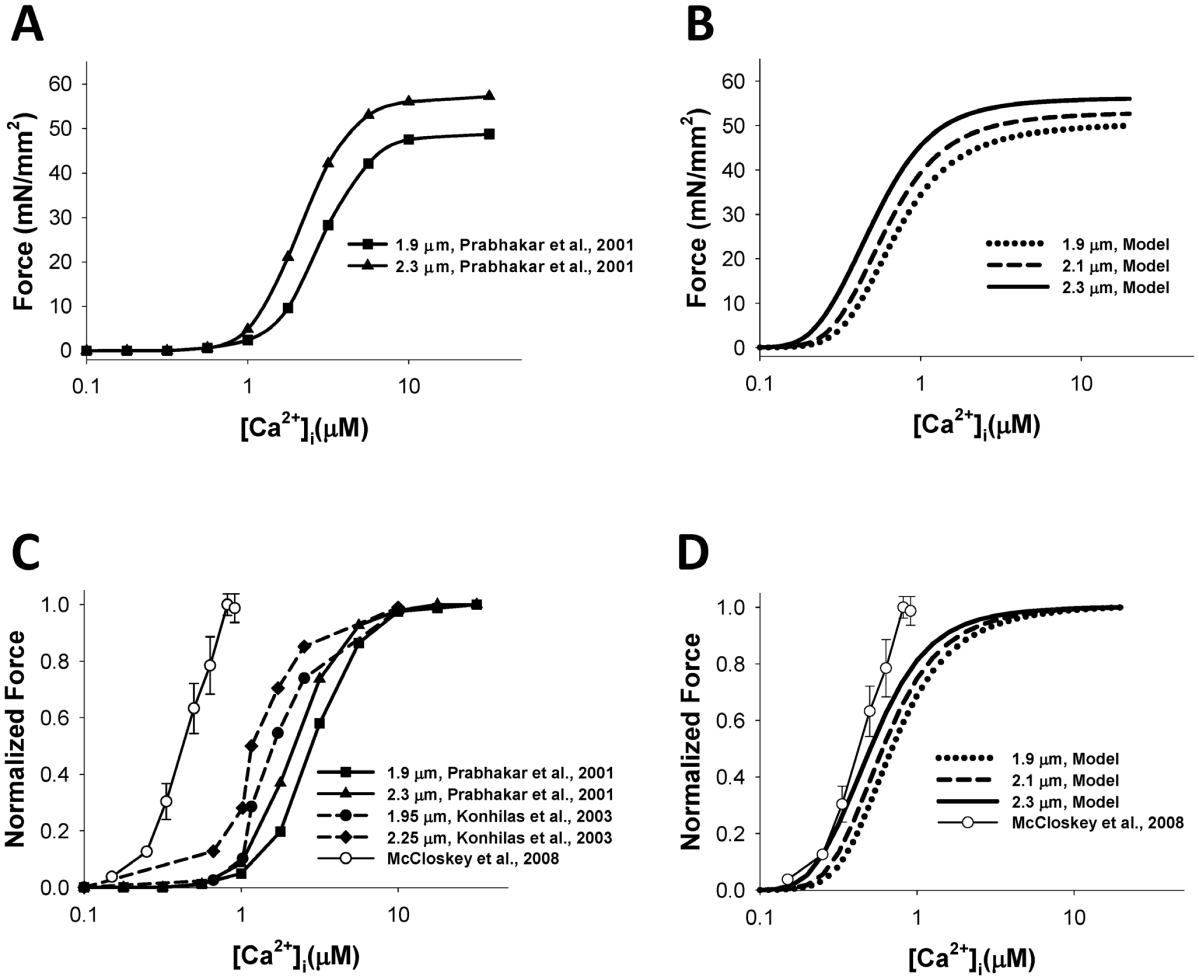
The steady-state [Ca^2+^]_i_-force relationship. The steady-state [Ca^2+^]_i_-absolute force relationship (A and B) and the normalized steady-state [Ca^2+^]_i_-force relationship (C and D). Experimental data from Prabhakar et al. [23] (SL = 1.9 and 2.3 µm) and Konhilas et al. [25] (SL = 1.95 and 2.25 µm), obtained with skinned myocytes, are shown in (A) and (C) with filled symbols; experimental data for non-skinned myocytes from [26] is shown by unfilled circles in (C) and (D). The model’s simulations at various initial sarcomere lengths (SL = 1.9, 2.1, and 2.3 µm) are shown in (B) and (D). Simulated data for both epicardial and endocardial cells are the same.

We first simulated steady-state force-calcium relationships. Both epicardial and endocardial cell models demonstrated the same simulation data for the steady-state force, as the contraction model Rice et al. [15] depends on intracellular [Ca^2+^]_I_ concentration. Figure 2A shows the Ca^2+^-dependence of the absolute value of contraction force obtained by Prabhakar et al. [23] for two different sarcomere lengths, 1.9 and 2.3 µm, from skinned mouse ventricular myocytes. For both cases, the force represents an increasing sigmoid function of calcium concentration. There is a relatively small increase in the saturation force from 48.8 to 57.2 mN/mm^2^ when sarcomere length increases by about 20%, from 1.9 to 2.3 µm. Figure 2B shows simulation of the steady-state force-calcium relationships for three sarcomere lengths, 1.9, 2.1, and 2.3 µm. Our model is able to closely reproduce the saturating value of the force for corresponding sarcomere lengths. However, there are some differences between simulated and experimental data in sensitivity to external Ca^2+^, as simulated force saturates at smaller values of Ca^2+^ concentrations. Such differences are due to a decrease in Ca^2+^ sensitivity of skinned compared to intact cardiac cells [24].

Our model is also able to reproduce a shift in Ca^2+^ sensitivity for steady-state force-calcium relationships shown for three sarcomere lengths (Fig. 2D). Such a shift can be clearly seen for normalized steady-state force-calcium relationships. Simulations show that an increase in sarcomere length leads to smaller half-saturation values of Ca^2+^ concentrations, demonstrating an increase in Ca^2+^ sensitivity (Fig. 2D). A similar shift in Ca^2+^ sensitivity is also observed experimentally for mouse cardiac cells (Fig. 2C) [23], [25].

In addition to the skinned mouse ventricular myocytes, our simulation data is also compared to the available experimental data on steady-state force-calcium relationships from intact cells, shown in Fig. 2C and 2D with unfilled circles [26]. Figure 2D shows that our simulations are in good agreement with the experimental data. IC_50_ and Hill coefficient *h* obtained by fitting steady-state force-calcium relationships from McCloskey et al. [26] data with the function
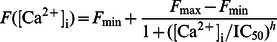
are 0.47 µM [Ca^2+^]_i_ and 3.05, respectively. Fitting our simulation data gives IC_50_ 0.68, 0.59, and 0.49 µM [Ca^2+^]_i_ and Hill coefficients 2.30, 2.33, and 2.25, for sarcomere lengths 1.9, 2.1, and 2.3 µm, respectively.

### Dynamic Behavior of Contraction Force

**Figure 3:**
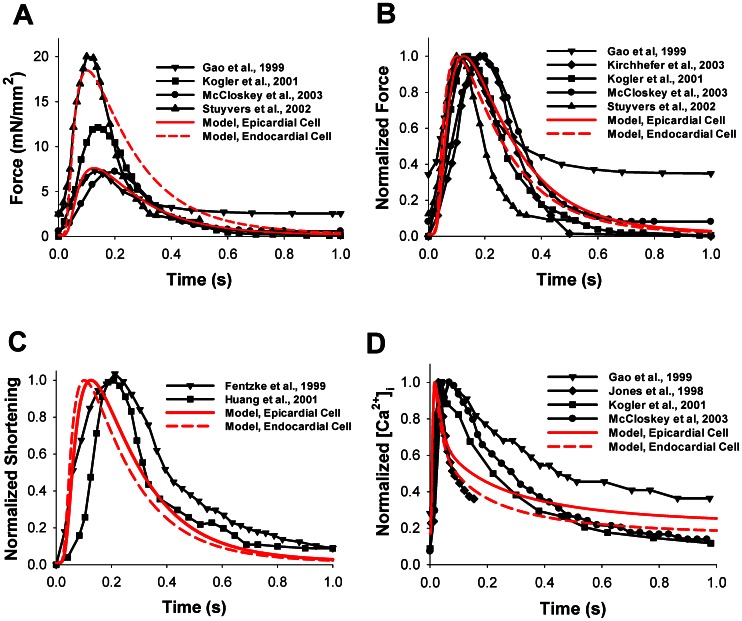
Contraction force, myocyte shortening, and [Ca^2+^]_i_ transients. The time course of force (in mN/mm^2^) (A) and normalized force (B) simulated by the model for epicardial (red solid lines) and endocardial (red dashed lines) cells are compared with experimental data from Stuyvers et al. [18], Gao et al. [27], Kirchhefer et al. [30], Kogler et al. [28], McCloskey et al. [29]. (C) Normalized shortening as a function of time. Simulation data is shown by red solid (epicardial cell) and red dashed (endocardial cell) lines, experimental data from Fentzke et al. [31] and Huang et al. [32] are shown by lines with symbols. (D) Normalized [Ca^2+^]_i_ transients as functions of time. The model simulation (red solid and red dashed lines for epicardial and endocardial cells, respectively) is compared to experimental data from Gao et al. [27], Jones et al. [44], Kogler et al. [28], and McCloskey et al. [29] (lines with symbols). For comparison, the initial sarcomere length in the model simulation is set to 2.1 µm, extracellular [Ca^2+^]_i_ concentration is 2 mM, and the frequency is 0.5 Hz, the frequency used most in the experimental data (see Table 1).

To test the ability of our model to reproduce the time behavior of the contraction force developed by mouse ventricular myocytes, we first stimulated the model cells with a constant frequency of 0.5 Hz. The time course of force in epicardial and endocardial cell simulations is plotted in Fig. 3A by red solid and dashed lines, respectively. As endocardial cells show larger [Ca^2+^]_i_ transients than epicardial cells, we obtained that the former develops stronger contraction force and larger shortening than the latter. The time behavior of the contraction forces obtained experimentally is shown by black solid lines with symbols [18], [27], [28], [29]. There are significant differences in the experimental data obtained from different experimental groups on the time behavior of force, both in peak values and residual forces (Table 1). Comparison of the time behavior of normalized simulated and experimental forces, both for epicardial and endocardial cells, shows a clear similarity in the time-to-peak values and relaxation of the simulated forces (Fig. 3B) [18], [27], [28], [29], [30].

**Table 1:** Experimental conditions for measurements of contraction force, cell shortening, and [Ca^2+^]_i_ transients and corresponding simulated conditions.

Reference	Temperature,°C	Sarcomere length, µm	[Ca^2+^]_o_, mM	Stimulation frequency, Hz	[Ca^2+^]_i_ indicator
Gao et al. 1999 [27]	20–22	2.1–2.2	2.0	0.5	Fura-2
Kirchhefer et al. 2003 [30]	Room	No data	2.0	0.5	
Kogler et al. 2001 [28]	22–23	2.1–2.2	2.0	0.5	Fura-2
McCloskey et al. 2003 [29]	22	2.1	2.0	0.5	Fura-2
Stuyvers et al. 2002 [18]	25	2.0–2.1	2.0	1.0	
Fentzke et al. 1999 [31]	22–23	2.3			
Huang et al. 2001 [32]	Room	2.3	0.5	0.5	
Jones et al. 1998 [44]	25		2.0		Fluo-3
Simulation, this paper	25	2.1	2.0	0.5	

Our model includes changes in sarcomere length during myocyte contraction. The time behavior of normalized sarcomere shortening for simulated cells is shown in Fig. 3C by red solid and dashed lines for epicardial and endocardial cells, respectively. The models do not show large differences in time-to-peak shortening and relaxation times. They closely reproduce myocyte shortening obtained in different experiments with mice (solid lines with symbols in Fig. 3C) [31], [32]. For comparison of the time scales of contraction force and Ca^2+^ dynamics, we also plot the time courses of the simulated and experimental intracellular Ca^2+^ transients by red lines and black solid lines with symbols in Fig. 3D, respectively. In each case, there is a delay in force development following the peak of the Ca^2+^ transient (compare times to peaks in Fig. 3B and 3D).

### Force-frequency Relationships

**Figure 4:**
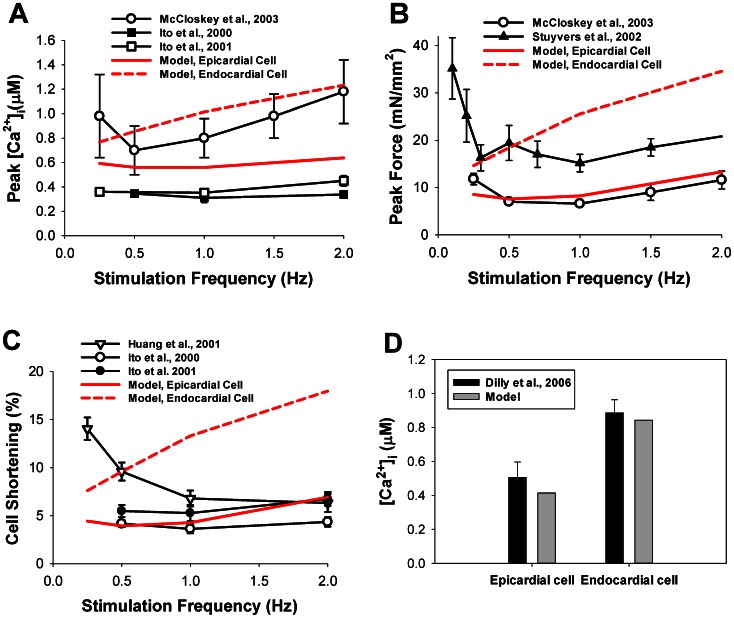
Stimulation frequency dependence of peak [Ca^2+^]_i_, peak force, and cell shortening. (A) Peak [Ca^2+^]_i_. (B) Peak force. (C) Cell shortening. The simulation data is shown by red solid (epicardial cell) and red dashed (endocardial cell) lines. The modeling results are compared to data from Ito et al. [33], [34] (A), McCloskey et al. [29] (A and B), and Huang et al. [32] (C). The initial SL for the simulation is 2.1 µm. (D) Experimental (black bars [35]) and simulated (gray bars) intracellular [Ca^2+^]_i_ transients obtained for epicardial and endocardial cells at stimulation frequency 1 Hz.

In order to investigate force-frequency relationships, we also stimulated model cells with different frequencies ranging from 0.25 to 2.0 Hz. Frequency dependences of intracellular Ca^2+^ transients, contraction force, and cell shortening are shown in Fig. 4. Our simulated peak [Ca^2+^]_i_-frequency relationship (red solid and dashed lines in Fig. 4a) is within the variability of experimental data (solid lines with symbols in Fig. 4A) [18], [29], [33], [34]. Note, that the simulated amplitudes of [Ca^2+^]_i_ transients for epicardial and endocardial cells are verified by the experimental data obtained by Dilly et al. [35] (Fig. 4D). The models are able to reproduce peak contraction force-frequency relationships for mouse ventricular myocytes in the frequency range from 0.5 to 2.0 Hz (Fig. 4B). The experimental data shows biphasic behavior of the peak force, with a decrease from 0.25 to 0.5–1.0 Hz, followed by an increase from 1.0 to 2.0 Hz [29], with a clear minimum in force-frequency relationships (however, see data of Ito et al. [34] were the minimum is less apparent). Our model reproduced such biphasic behavior of the force-frequency relationships for epicardial cells. Peak contraction force for endocardial cells increases with stimulation frequency.

Finally, we are able to simulate peak lengthening-frequency relationships (red lines in Fig. 4C). While some experimental data shows consistent decrease in cellular shortening with frequency [32], other data follows biphasic behavior [33], [34] (solid lines with symbols in Fig. 4C). Our modeling data demonstrates biphasic behavior in cell shortening for epicardial cells, which is consistent with the biphasic behavior of the contraction force and [Ca^2+^]_i_ transients (red solid lines in Fig. 4A, 4B, and 4C). Model endocardial cells show only an increase in cell shortening as well as in [Ca^2+^]_i_ (red dashed lines in Fig. 4A and 4C, respectively).

**Figure 5:**
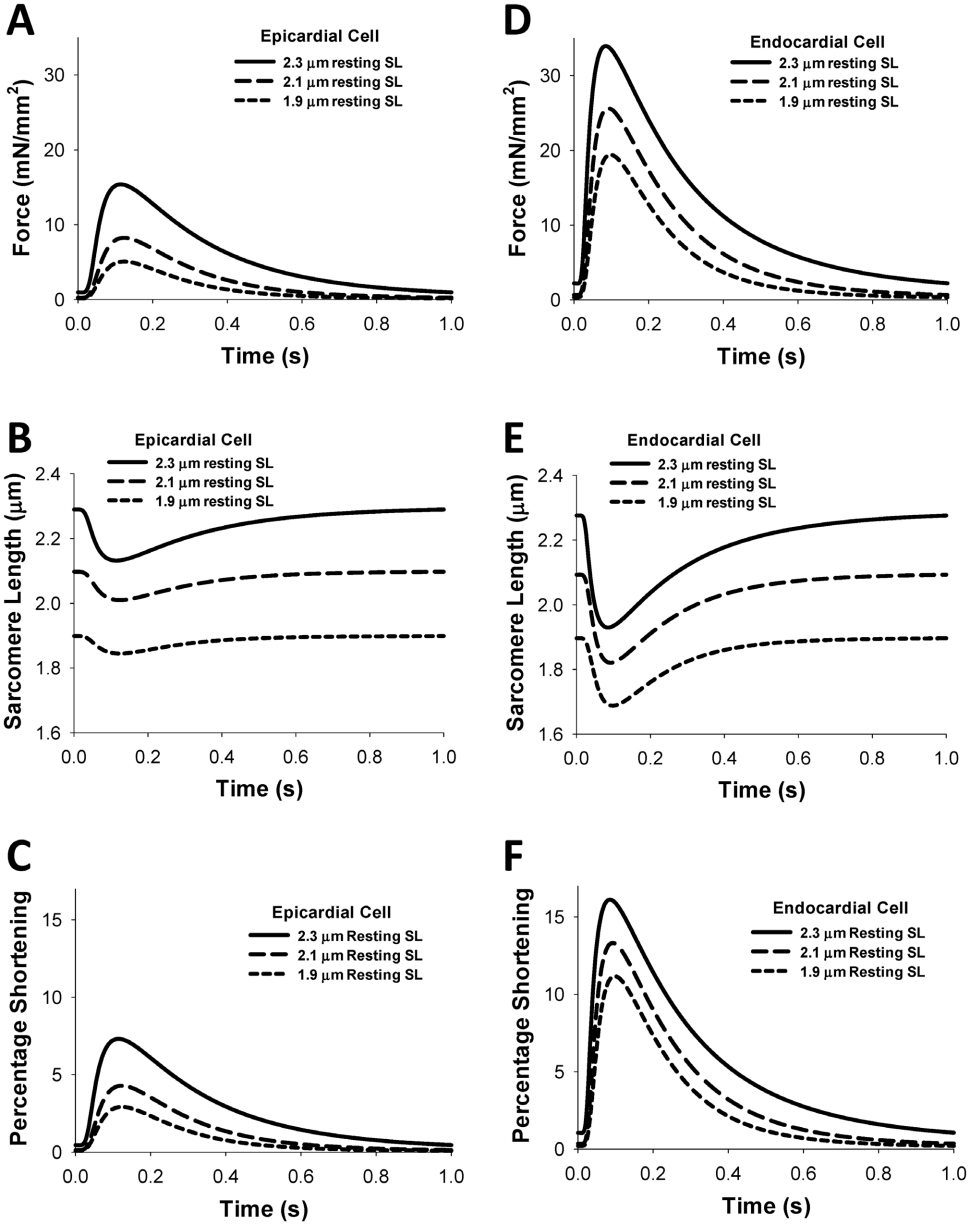
Time course of the contraction force, sarcomere length, and percentage of shortening for epicardial and endocardial cells for different sarcomere lengths. Simulations with different resting sarcomere lengths (SL_0_ = 1.9, 2.1, and 2.3 µm) show a significant difference in the magnitude of the contraction force (A, D), sarcomere length (B, E) and percentage of sarcomere length shortening (C, F). The stimulation frequency for each simulation is 1 Hz. Simulations are performed for epicardial (A, B, C) and endocardial (D, E, F) cells.

Simulated time courses for contraction forces, sarcomere lengths, and sarcomere shortenings for three different resting sarcomere lengths (1.9, 2.1, and 2.3 µm) for epicardial and endocardial cells are shown in Fig. 5. As seen from the figure, an increase in the resting sarcomere length increases twitch force and relative sarcomere shortening. Similar behavior is also observed experimentally and from the simulations of others [9], [11]. At comparable sarcomere lengths, the endocardial cells develop larger contraction force and sarcomere shortening than the epicardial cells (Fig. 5).

### Constant versus Variable Sarcomere Length

While steady-state simulations show that peak force is dependent on the initial sarcomere length, there is also a dynamic relationship between force and sarcomere length. Our models use a variable SL when calculating the transition rate from non-permissive to permissive states, as well as in the detachment rates in permissive states. To see the effect of using a variable SL in the transition rate equations, we ran simulations in which a constant SL replaced the variable SL in the calculation of the normalized sarcomere length
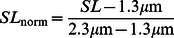
(11)which is used in the detachment rates and transition rates in Markov model (Fig. 1B)



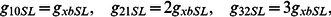
(12)

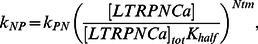
(13)

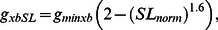
(14)

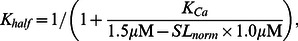
(15)

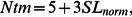
(16)where K_Ca_ = k^−^_ltrpn_/k^+^_ltrpn_, and the constants can be found in the Supporting Information (Appendix S1).

**Figure 6:**
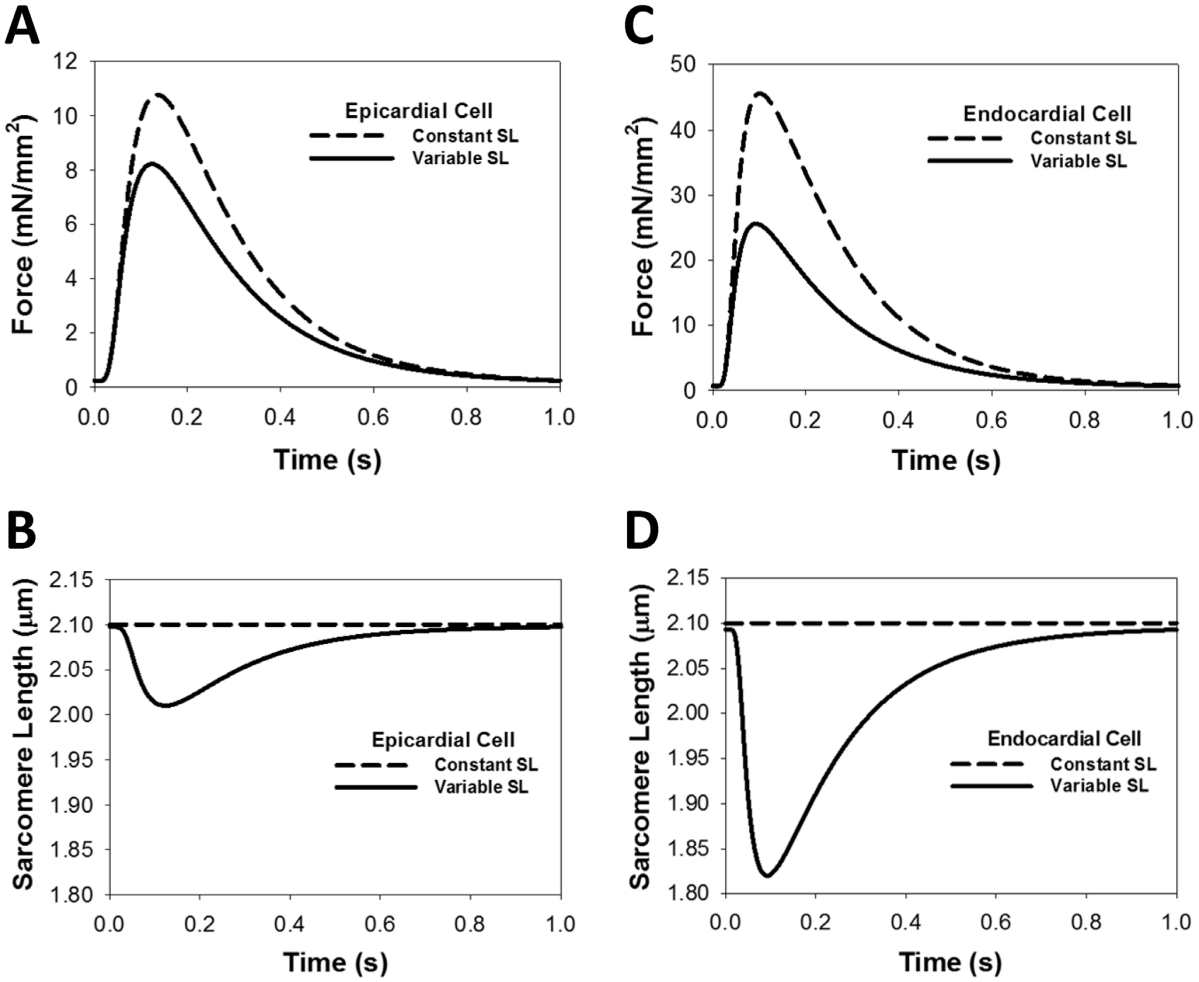
The effects of constant and variable sarcomere lengths on the contraction force development and myocyte shortening. (A) Force development for the models with variable (solid line) and constant (dashed line) sarcomere lengths. Changing the SL from a variable to a constant (B, D) does not change [Ca^2+^]_i_ transients, but changes contraction force (A, C). The initial SL for each simulation is 2.1 µm with a stimulation frequency of 1 Hz. Simulation data shows an increase in force, both for epicardial (A) and endocardial (C) cells, when variable SL is replaced by constant SL.

**Figure 7:**
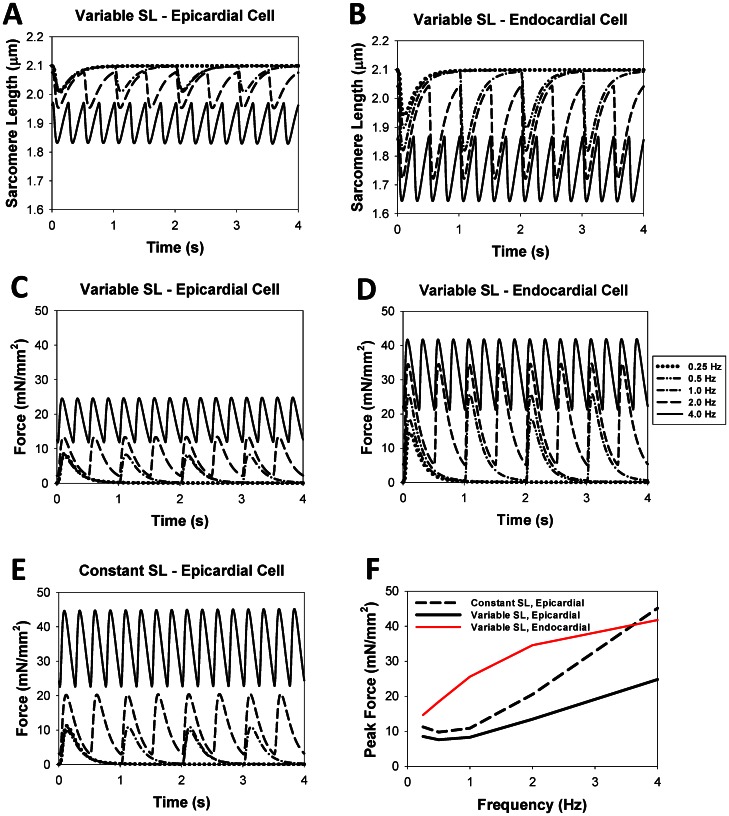
The effects of stimulation frequency on the time behavior of sarcomere length and contraction force for epicardial and endocardial cells. The time courses of the SL (A and B) and contraction force (C, D, and E) over a four second interval are shown at different stimulation frequencies for epicardial (A, C, and E) and endocardial (B and D) cells. The simulation data with constant SL is shown only for epicardial cells, as the data for endocardial cells is similar. The frequency dependence of force for an epicardial cell when a variable SL parameter is used is not as pronounced as the frequency dependence of force when a constant SL parameter is used (C and E). The initial SL for each simulation is 2.1 µm, but the residual force for higher frequencies leads to significant shortening (A and B). Frequency dependence of peak force for epicardial and endocardial cells with variable SL and for epicardial cells with constant SL is shown in (F).

Figure 6A shows force development in epicardial cells at a stimulation rate of 1 Hz in the simulation using a constant SL (dashed line) versus the simulation using a variable SL (solid line) (see also Fig. 6B). Data for endocardial cells displays similar behavior and is shown in Fig. 6C and 6D. The peak force when using a constant SL is clearly higher, while the residual force appears to be about the same. However, simulations run at various frequencies show that the peak and residual force when using a constant SL (Fig. 7E) is always higher than corresponding forces when a variable SL is used (Fig. 7C). Even though there is a difference in the magnitude of force, the frequency dependence of peak force when using a constant SL (black dashed line in Fig. 7F) is similar to the frequency dependence when a variable SL is used (black solid line in Fig. 7F). For comparison, Figs. 7B and 7D show simulated data on cell shortening and contraction force at different stimulation frequencies for endocardial cells, using variable sarcomere length (data on constant SL is not shown). As seen from the figures, both peak contraction force and cell shortening are larger for the endocardial cells than the epicardial cells.

In both cases, constant and variable SL, we observed a decrease in time-to-peak and time to 50% relaxation rate for the contraction force with an increase of stimulation frequency starting from 0.5 Hz. A similar increase in the residual contraction force at the larger stimulation frequencies is also observed experimentally [36].

### Frequency Dependence of dL/dt and dF/dt

**Figure 8:**
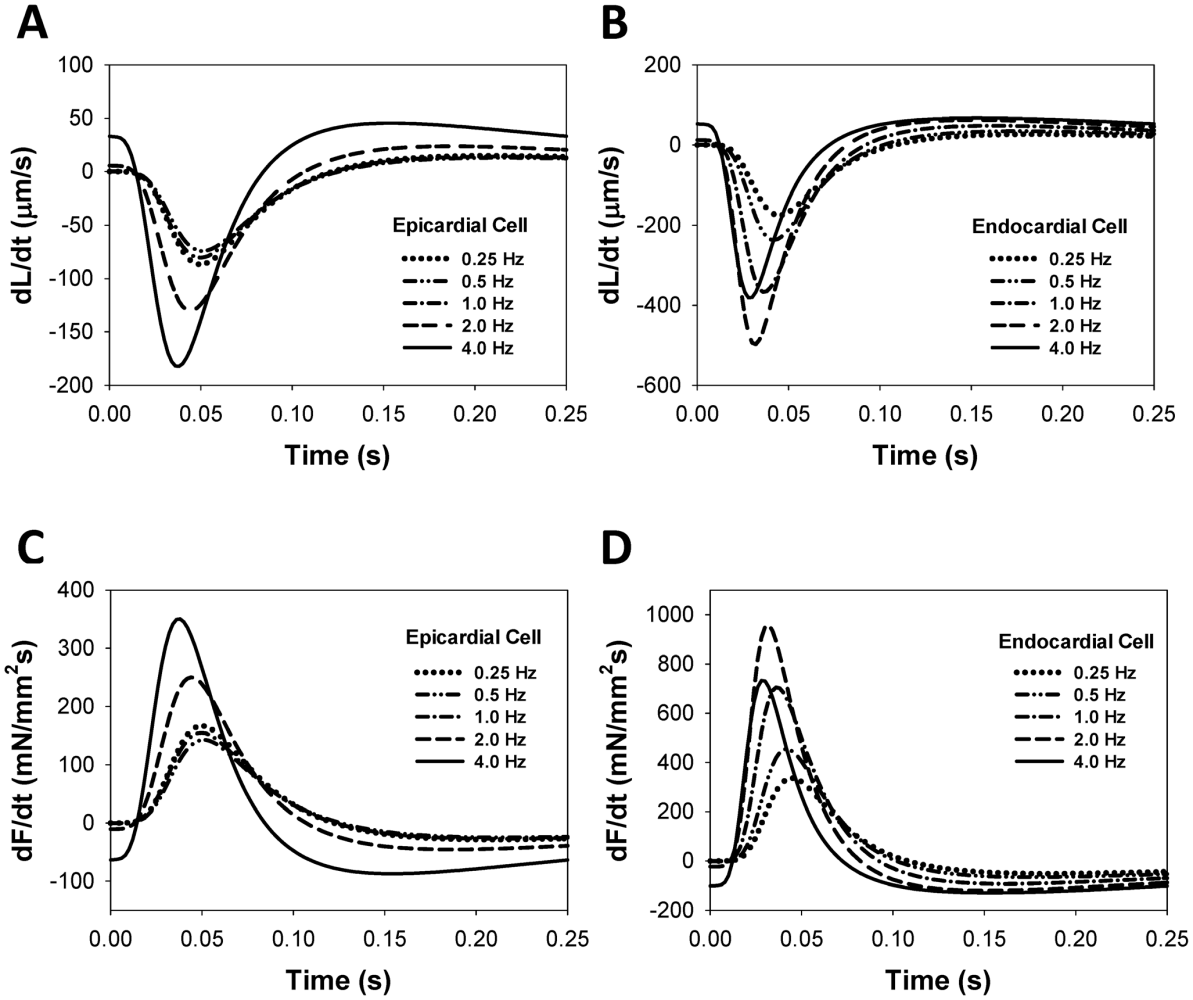
Time behavior of dL/dt and dF/dt for different stimulation frequencies. Simulated time course of the rates of cellular shortening dL/dt (A, B) and contraction force dF/dt (C, D) during twitches for epicardial (A, C) and endocardial (B, D) cells. For epicardial cells, the largest values of ±dL/dt_max_ and ±dF/dt_max_ are observed at relatively fast stimulation frequency of 4 Hz (solid lines in (A) and (C)). For endocardial cells, the largest values of ±dL/dt_max_ and ±dF/dt_max_ occur in the frequency interval from 1 to 4 Hz (dashed lined in (B) and (D)).

The frequency dependencies of the peak force and cell shortening are shown in Fig. 4. As might be expected, dL/dt and dF/dt also show frequency dependence. Simulated time courses for dL/dt (Fig. 8A and 8B) and dF/dt (Fig. 8C and 8D) are shown for various frequencies from 0.25 Hz to 4.0 Hz, both for epicardial (Fig. 8A and 8C) and endocardial (Fig. 8B and 8D) cells. A negative dL/dt value indicates cell shortening during a contraction, while a positive dL/dt corresponds to relaxation. A positive dF/dt indicates the increase in force during a contraction, while a negative dF/dt corresponds to relaxation. The epicardial cell demonstrates a monotonic increase in the magnitudes of peak values for dL/dt and dF/dt in the frequency range from 0.25 to 4 Hz (Fig. 8A and 8C). In contrast, the endocardial cell shows a biphasic behavior in the peak magnitudes of the derivatives: an increase when the stimulation frequency changes from 0.25 to 2 Hz, and a decrease in the frequency range from 2 to 4.0 Hz (Fig. 8B and 8D).

**Figure 9:**
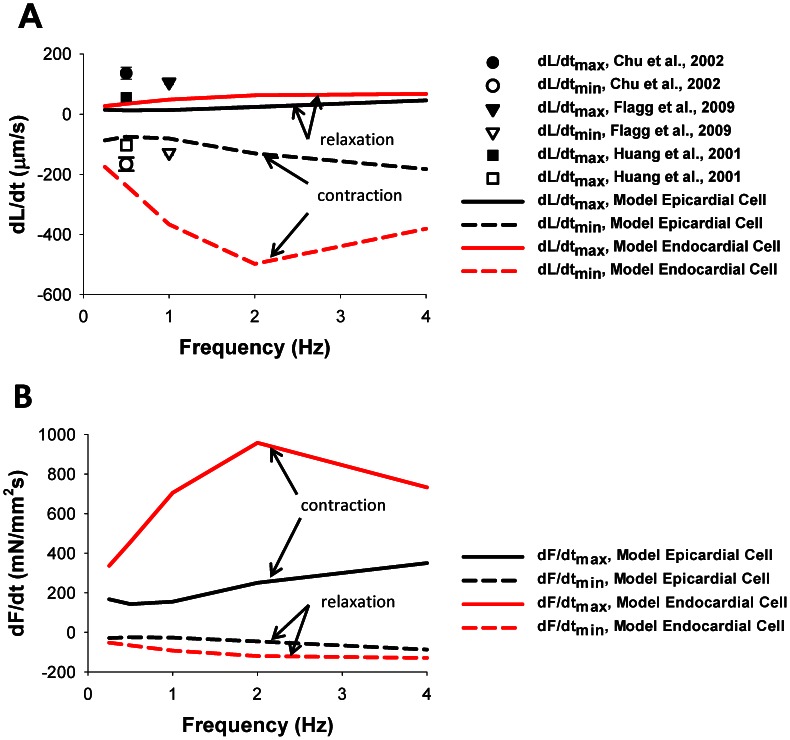
Frequency dependence of dL/dt_max_ and dF/dt_max_. (A) The simulated frequency dependences of (+dL/dt)_max_ (solid lines) and (−dL/dt)_max_ (dashed lines). Experimental data from Chu et al. [37], Flagg et al. [38], and Huang et al. [32] are shown by symbols. We consider (−dL/dt) to correspond to cell shortening. (B) The simulated frequency dependence of (+dF/dt)_max_ (solid lines) and (−dF/dt)_max_ (dashed lines). We consider (+dF/dt) to correspond to contraction. The initial SL for the simulations in (A) and (B) is 2.1 µm. Data for epicardial and endocardial cells are shown in black and red, respectively.

The frequency relationship for +dL/dt_max_ (solid lines) and −dL/dt_max_ (dashed lines) is shown in Fig. 9A. Both values show biphasic behavior. For the epicardial cell, +dL/dt_max_ and −dL/dt_max_ decrease at stimulation frequencies from 0.25 to 0.5 Hz, and then increase for stimulations frequencies up to 4 Hz. For the endocardial cell, +dL/dt_max_ and −dL/dt_max_ increase at stimulation frequencies from 0.25 to 2.0 Hz, and then decrease for stimulations frequencies up to 4 Hz. When compared to the experimental data, our model tended to show, on average, peak contraction rates approximately equal to experimental data (open symbols) [32], [37], [38]. However, the model showed somewhat slower relaxation, thus lower values of +dL/dt_max_, than experimental data (solid symbols) [32], [37], [38].

Figure 9B shows the frequency relationship for +dF/dt_max_ (solid lines) and −dF/dt_max_ (dashed lines). As with corresponding values for +dL/dt_max_ and −dL/dt_max_, the +dF/dt_max_ and −dF/dt_max_ show biphasic behavior for both epicardial and endocardial cells.

**Figure 10:**
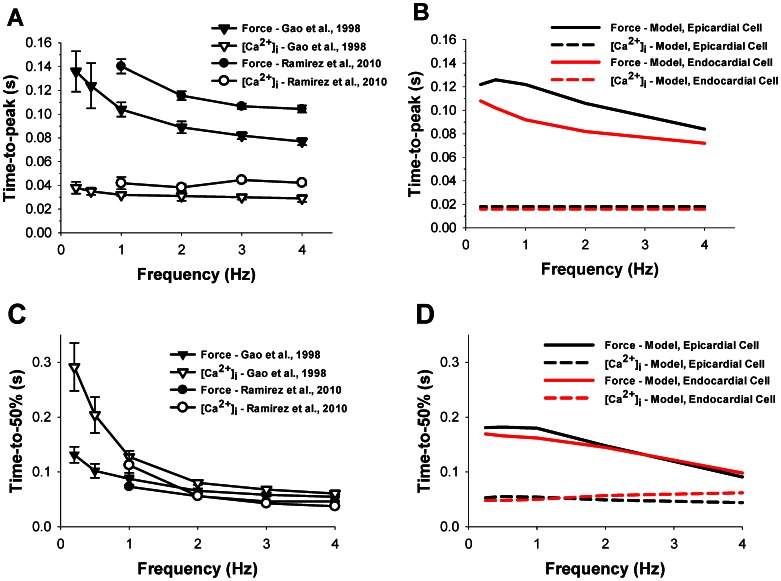
Time-to-peak and time-to-50% relaxation of the contraction force and [Ca^2+^]_i_ transients as function of stimulation frequency. Experimental (A) and simulated (B) frequency dependencies of time-to-peaks for intracellular [Ca^2+^]_i_ transients and contraction force, and experimental (C) and simulated (D) frequency dependencies of time-to-50% relaxations for intracellular [Ca^2+^]_i_ transients and contraction force. Experimental data are obtained by Gao et al. ([27], triangles) and Ramirez et al. ([45], circles). Unfilled and filled symbols are used for intracellular [Ca^2+^]_i_ transients and contraction force, respectively. Simulation data for contraction force and intracellular [Ca^2+^]_i_ transients are shown by solid and dashed lines, respectively, and data for epicardial and endocardial cells are shown in black and red, respectively.

To compare experimental and simulated data quantitatively, we plotted experimental and simulated results on time-to-peak and time-to-50% relaxation of the contraction force and intracellular [Ca^2+^]_i_ transients in Fig. 10. Simulated data are shown for both epicardial and endocardial cells (black and red, respectively, in Fig. 10B and 10D). Simulated data for time-to-peak force shows good agreement with the experimental data (compare Fig. 10B and 10A), while time-to-50% relaxation are somewhat longer in the simulated data than those obtained in the experiments (compare Fig. 10D and 10C). Experimental data for time-to-peak and time-to-50% relaxation of [Ca^2+^]_i_ transients are somewhat longer than those from simulations, but the simulated time-to-50% relaxations approach the experimental values at larger frequencies. Epicardial and endocardial cells show similar simulated values for time-to-peak and time-to-50% relaxation of [Ca^2+^]_i_ transients, and for time-to-50% relaxation of contraction force. However, there are moderate differences between the cells for time-to-peak of the contraction force (Fig. 10B).

## Discussion

In this paper, we developed a new model for mouse ventricular myocyte contraction. This model is based on our previously published models for epicardial and endocardial cells [19], [20], [21], [22], which includes a comprehensive description of action potential, ionic currents, and Ca^2+^ dynamics. For a description of myocyte contraction, we adopted Model 4 developed by Rice et al. [15] by fitting experimental data on contraction for mice.

Mice demonstrate considerably faster heartbeats than many other species. Their contraction rate is about 10 beats per second [39], which is, for example, faster than the rabbit (4 Hz, [40]) and human (1 Hz, [41]) heart contraction rates. In addition, the action potential duration in mouse ventricular myocytes is also considerably shorter (APD_50_ ∼ 4.5 ms in mice [19] versus ∼200 ms in rabbits [9] and ∼300–400 ms in humans [42]). These differences suggest different time characteristics for contractions in mouse, compared to human or rabbit, ventricular myocytes.

**Figure 11:**
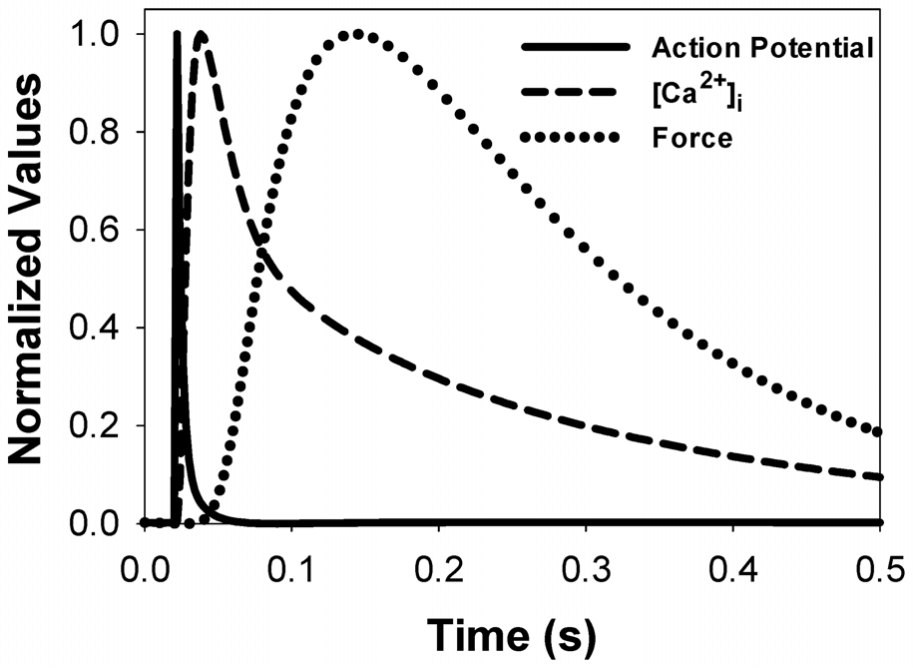
Scaling action potential, [Ca^2+^]_i_ transient, and force in the model for mouse ventricular myocyte contraction. The simulation used an initial SL of 2.1 µm and a stimulation frequency of 1 Hz. In mouse ventricular myocytes, Ca^2+^ transient develops after action potential repolarization is almost complete. After that, the contraction force develops with time delay due to Ca^2+^ binding to troponin and troponin-induced changes in contractile proteins.

In a mouse cardiac cell, at moderate stimulation rates, an increase in action potential is followed by an increase in [Ca^2+^]_i_ and a delayed increase in force. The peak value of Ca^2+^ transient occurs after almost complete repolarization of action potential. In addition, peak contraction force appears after a significant decline of [Ca^2+^]_i_. Our model replicates this relationship. Figure 11 shows normalized values for epicardial action potential (solid line), [Ca^2+^]_i_ (dashed line), and force (dotted line) over a 0.5 second interval for a simulation at 1 Hz. In larger species, such as rabbit, time scaling of the action potential, [Ca^2+^]_i_ and contraction force transients is different (Fig. 9 in [9]). For rabbits, [Ca^2+^]_i_ transient, in significant part, overlaps with the action potential and contraction force transient, while the peak sequence is the same as in mice.

Mouse ventricular myocytes, unlike other species, demonstrate biphasic frequency dependence of intracellular [Ca^2+^]_i_ transient and peak force [18], [29] (however, see data of Ito et al. [34] were biphasic behavior is less apparent). Stuyvers et al. [18] suggested a qualitative mechanism which explains this biphasic behavior based on frequency-dependent Ca^2+^ dynamics. The minimum occurs at the crossroad of the descending frequency trend of the Ca^2+^ load into the sarcoplasmic reticulum during diastole and ascending trend in Ca^2+^ entry into the cell through L-type Ca^2+^ channels. They used a simplified description of Ca^2+^ dynamics for mouse ventricular myocytes. Our model for an epicardial cell, which includes a comprehensive description of the electrical activity and Ca^2+^ dynamics in mouse myocytes during cell twitch, was also able to reproduce this physiological phenomenon. In our model, myocyte contraction force is related to Ca^2+^ dynamics through the Markov model for crossbridge kinetics. While both peak [Ca^2+^]_i_ transients and peak contraction force show minimum values as functions of stimulation frequency, these minimum frequency values are slightly different (Fig. 4). This trend is also confirmed by the experimental data of McCloskey et al. [29].

However, our model for the endocardial cell does not show biphasic behavior in the frequency-dependence of both peak [Ca^2+^]_i_ transients and peak contraction force. There are also some experimental data in which non-monotonic increase in peak [Ca^2+^]_i_ transients and myocyte shortening in mice is less apparent: even saturation and decrease in myocyte shortening amplitude at relatively large stimulation frequencies occur [34]. Our model for the endocardial cell, at least qualitatively, reproduced saturation and even decrease in sarcomere shortening and contraction force amplitude at 4-Hz stimulation (Fig. 7B and 7D). This effect can be explained by the larger peak and diastolic values of [Ca^2+^]_i_ transients in endocardial cells compared to epicardial cells, which shift the operation interval of intracellular Ca^2+^ towards a smaller slope in force-calcium relationships (Fig. 2D).

While there are no specific experimental studies of contraction force and cell shortening in mouse epicardial and endocardial ventricular myocytes, there are a few studies of the differences in action potentials and Ca^2+^ handling in these cells [35], [43]. The studies show that the endocardial cells demonstrate significantly larger [Ca^2+^]_i_ transients, and our modeling predicts larger contraction force and shortening in these ventricular myocytes.

Our electromechanical model for mouse ventricular myocyte contraction includes a variable sarcomere length during cell contraction, the effect that occurs in most experiments. Simulations with variable sarcomere length produce significantly smaller contraction force than the simulations with constant sarcomere length despite the same time course and amplitude of [Ca^2+^]_i_ transient during twitch. This suggests the importance of the inclusion of cell shortening in the model for cardiac myocyte contraction. Note that a similar result was obtained with a more complex model of Rice et al. [9], developed for rabbit ventricular myocytes, who also studied the effects of variable and fixed sarcomere length on the force development.

Several models for cardiac myocyte contraction have been developed to date [7], [9], [11], [15], [17] (see also review [13]). Earlier models did not include sarcomere shortening during twitch [7], [15], [17]. They are primarily focused on simplification of the description of crossbridge kinetics, their dependence on Ca^2+^ dynamics, and careful reproduction of the existing experimental data on steady-state and dynamic force-calcium relationships. Most of these models have limitations due to this and other simplifications.

Rice et al. [15] investigated five Markov models describing contraction mechanisms in cardiac myocytes. Two of the models consisted of four tropomyosin states and transitions between them (N0, N1, P0, and P1, see Fig. 1B). These models differ by the mechanisms of modulation of the transition rates (in Fig. 1B they are defined as k_NP_ and k_PN_). In Model 1, rates k_NP_ and k_PN_ are independent of the developed force, while in Model 2 the rates depend on the developed force. In both models, Ca^2+^ binding to troponin directly affects tropomyosin shifting, i.e., rates k_NP_ and k_PN_. Model 3 includes an indirect connection of the Ca^2+^ binding to troponin and tropomyosin shifting, as shown by dashed arrows in Fig. 1B (see also [15]), and only four states (N0, N1, P0, and P1). Models 4 and 5 were extended to up to three crossbridge bindings, which resulted in four permissive tropomyosin states, P0, P1, P2, and P3, (Fig. 1B and [15]). The only difference between Models 4 and 5 is the modulation of the k^−^_ltrpn_ rate by generated force. Because Model 4 and Model 5 yielded an approximately equal description of myocyte contraction, we implemented Model 4 in our electrophysiological model, as Model 5 led to unstable solutions.

Our model of mouse ventricular myocyte contraction also has some limitations due to the simplification of the biophysical mechanism of contraction. In particular, the model uses a simplified description of the relationships between contraction force and cellular shortening in the form of Hook’s law, while the real dependence is more complicated [9]. It does not describe the effects of cellular shortening on Ca^2+^ transients, as does the model of Rice et al. [9]; however, this effect is relatively small. Also, our model, as most other models, did not take into account intracellular spatial inhomogeneities of Ca^2+^ concentration and crossbridge binding sites.

Nevertheless, despite the limitations, our electromechanical model of mouse ventricular myocyte contraction was extensively verified by experimental data obtained for mice. It reproduced reasonably well a significant amount of the existing experimental data. The model can be used for cells from two different regions of the heart (epicardium and endocardium). As with most other models, it uses a simplified description of the contraction force generation. We employed a six-state Markov model for tropomyosin dynamics and separate Ca^2+^ binding to troponin (Fig. 1B) to describe force development. More comprehensive models will be necessary to develop a better simulation of more extended experimental data sets.

## Supporting Information

Appendix S1 **Model Summary.**
(DOC)
