# Supplementary material for: A Mathematical Model of the Mouse Ventricular Myocyte Contraction
Source: PLoS One. 2013 May 9;8(5):e63141. doi: 10.1371/journal.pone.0063141 (PMC3650013; doi:10.1371/journal.pone.0063141)
Supplement: Appendix S1 — Model Summary. (DOC) [file pone.0063141.s001.doc]

**APPENDIX: MODEL SUMMARY**

1. **Membrane Potential**

$$\begin{matrix} \frac{dV}{dt}=-\frac{1}{C_{m}}(I_{CaL}+I_{p(Ca)}+I_{NaCa}+I_{Cab}+I_{Na}+I_{Nab}+I_{NaK}+I_{Kto,f} \\ +I_{Kto,s}+I_{K1}+I_{Ks}+I_{Kur}+I_{Kss}+I_{Kr}+I_{Cl,Ca}-I_{stim}) \end{matrix}$$

(A .1)

**2. Calcium Dynamics**

$\frac{d[Ca^{2+}]_{i}}{dt}=B_{i}\left\{J_{leak}+J_{xfer}-J_{up}-J_{trpn}-\left(I_{Cab}-2I_{NaCa}+I_{p(Ca)}\right)\frac{A_{cap}C_{m}}{2V_{myo}F}\right\}$**2.1. Calcium Concentration**

(A .2)

(A .3)

(A .4)

(A .5)

(A .6)

(A .7)

(A .8)

**2.2. Calcium Fluxes**

(A .9)

(A .10)

(A .11)

(A .12)

(A .13)

(A .14)

(A .15)

**2.3. Calcium Buffering**

(A .16)

(A .17)

$\frac{d[LTRPNCa]}{dt}=k_{ltrpn}^{+}[Ca^{2+}]_{i}\left\{[LTRPN]_{tot}-[LTRPNCa]\right\}-k_{ltrpn}^{-}[LTRPNCa]$

**2.4. Ryanodine Receptors**

$\frac{dP_{O1}}{dt}=k_{a}^{+}[Ca^{2+}]_{ss}^{n}P_{C1}-k_{a}^{-}P_{O1}-k_{b}^{+}[Ca^{2+}]_{ss}^{m}P_{O1}+k_{b}^{-}P_{O2}-k_{c}^{+}P_{O1}+k_{c}^{-}P_{C2}$

(A .18)

(A .19)

(A .20)

(A .21)

$\frac{dP_{C2}}{dt}=k_{c}^{+}P_{O1}-k_{c}^{-}P_{C2}$

**2.5. Calcium Currents**

*2.5.1. L-type Calcium Current*

$$I_{CaL}=G_{CaL}O(V-E_{Ca,L})$$

(A .22)

(A .23)

(A .24)

(A .25)

(A .26)

(A .27)

(A .28)

(A .29)

(A .30)

(A .31)

(A .32)

(A .33)

(A .34)

*2.5.2. Calcium Pump Current*

(A .35)

(A .36)

*2.5.3. Na^+^-Ca^2+^ Exchange Current*

$$\begin{matrix} I_{NaCa}=k_{NaCa}\frac{1}{K_{m,Na}^{3}+[Na^{+}]_{0}^{3}}\frac{1}{K_{m,Ca}+[Ca^{2+}]_{0}}\frac{1}{1+k_{sat}e^{(\eta-1)VF/RT}} \\ \\ \times(e^{\eta VF/RT}[Na^{+}]_{i}^{3}[Ca^{2+}]_{0}-e^{(\eta-1)VF/RT}[Na^{+}]_{0}^{3}[Ca^{2+}]_{i}) \end{matrix}$$

*2.5.4. Calcium Background Current*

(A .37)

(A .38)

**3. Sodium Dynamics**

(A .39)

**3.1. Sodium Concentration**

$$\frac{d[Na^{+}]_{i}}{dt}=-(I_{Na}+I_{Nab}+3I_{NaCa}+3I_{NaK})\frac{A_{cap}C_{m}}{V_{myo}F}$$

**3.2. Fast Sodium Current**

(A .40)

(A .41)

(A .42)

(A .43)

(A .44)

(A .45)

(A .46)

(A .47)

(A .48)

(A .49)

(A .50)

(A .51)

(A .52)

(A .53)

(A .54)

(A .55)

(A .56)

(A .57)

(A .58)

(A .59)

(A .60)

(A .61)

(A .62)

(A .63)

(A .64)

**3.3. Background Sodium Current**

(A .65)

$I_{Nab}=G_{Nab}(V-E_{Na})$

**4. Potassium Dynamics**

**4.1. Potassium Concentration**

$$\frac{d[K^{+}]_{i}}{dt}=-(I_{Kto,f}+I_{Kto,s}+I_{K1}+I_{Ks}+I_{Kss}+I_{Kur}+I_{Kr}-2I_{NaK})\frac{A_{cap}C_{m}}{V_{myo}F}$$

(A .66)

**4.2. Transient Outward Potassium Current *I_Kto,f_***

$$I_{Kto,f}=G_{Kto,f}a_{to,f}^{3}i_{to,f}(V-E_{K})$$

(A .67)

(A .68)

(A .69)

(A .70)

(A .71)

(A .72)

(A .73)

(A .74)

**4.3. Transient Outward Potassium Current *I_Kto,s_***

$$I_{Kto,s}=G_{Kto,s}a_{to,s}i_{to,s}(V-E_{K})$$

(A .75)

(A .76)

(A .77)

(A .78)

(A .79)

(A .80)

(A .81)

$$\tau_{ti,s}=270.0+\frac{1050.0}{1+e^{(V+45.2)/5.7}}$$

**4.4. Time-independent Potassium Current**

(A .82)

$$I_{K1}=0.3397\left(\frac{[K^{+}]_{0}}{[K^{+}]_{0}+210.0}\right)\left(\frac{V-E_{K}}{1+e^{0.0448(V-E_{K})}}\right)+0.0193V$$

**4.5. Slow Delayed-Rectifier Potassium Current**

(A .83)

(A .84)

(A .85)

(A .86)

## 4.6. Ultra-Rapidly Activating Delayed-Rectifier Potassium Current

$$I_{Kur}=G_{Kur}a_{ur}i_{ur}(V-E_{K})$$

(A .87)

(A .88)

(A .89)

(A .90)

(A .91)

(A .92)

## 4.7. Non-Inactivating Steady-State Potassium Current

$$I_{Kss}=G_{Kss}a_{Kss}(V-E_{K})$$

$$\frac{da_{Kss}}{dt}=\frac{a_{ss}-a_{Kss}}{\tau_{Kss}}$$

(A .93)

(A .94)

(A .95)

**4.8. Rapid Delayed Rectifier Potassium Current (*m*ERG)**

$$I_{Kr}=O_{K}\cdot G_{Kr}\cdot\left[V-\frac{RT}{F}\ln\left(\frac{0.98[K^{+}]_{o}+0.02[Na^{+}]_{o}}{0.98[K^{+}]_{i}+0.02[Na^{+}]_{i}}\right)\right]$$

(A .96)

(A .97)

(A .98)

(A .99)

(A .100)

(A .101)

(A .102)

(A .103)

(A .104)

(A .105)

(A .106)

(A .107)

**5. Sodium-Potassium Pump Current**

$$I_{NaK}=I_{NaK}^{\max}f_{NaK}\frac{1}{1+(K_{m,Nai}/[Na^{+}]_{i})^{3/2}}\frac{[K^{+}]_{0}}{[K^{+}]_{0}+K_{m,Ko}}$$

(A .108)

(A .109)

(A .110)

**6. Calcium-Activated Chloride Current**

$$I_{Cl,Ca}=G_{Cl,Ca}O_{Cl,Ca}\frac{[Ca^{2+}]_{i}}{[Ca^{2+}]_{i}+K_{m,Cl}}(V-E_{Cl})$$

(A .111)

(A .112)

**7. Contraction**

**7.1 Permissive states**

$$\frac{dN0}{dt}=k_{PN}P0+g_{10SL}N1-k_{NP}N0$$

(A .113)

(A .114)

(A .115)

(A .116)

(A .117)

(A .118)

(A .119)

(A .120)

(A .121)

(A .122)

(A .123)

(A .124)

(A .125)

(A .126)

(A .127)

(A .128)

(A .129)

(A .130)

(A .131)

**7.2 Contraction Force**

(A .132)

(A .133)

(A .134)

(A .135)

(A .136)

(A .137)

(A .138)

$$\Sigma=\left(g_{\min xb}\right)\left(2g_{\min xb}\right)\left(3g_{\min xb}\right)+f_{01}\left(2g_{\min xb}\right)\left(3g_{\min xb}\right)+f_{01}f_{12}\left(3g_{\min xb}\right)+f_{01}f_{12}f_{23}$$

**8. Model parameters**

**Cell Geometry Parameters**

Parameter Definition Value

*A_cap_* Capacitive membrane area 1.534 × 10^-4^ cm^2^

*V_myo_* Myoplasmic volume 25.84 × 10^-6^ μL

*V_JSR_* Junctional SR volume 0.12 × 10^-6^ μL

*V_NSR_* Network SR volume 2.098 × 10^-6^ μL

*V_ss_* Subspace volume 1.485 × 10^-9^ μL

**Extracellular Ion Concentrations**

Parameter Definition Value

[*K^+^*]*_o_* Extracellular K^+^ concentration 4,000 μM

[*Na^+^*]*_o_* Extracellular Na^+^ concentration 136,000 μM

[*Ca^2+^*]*_o_* Extracellular Ca^2+^ concentration 2,000 μM

**SR Parameters**

Parameter Definition Value

*v_1_* Maximum RyR channel Ca^2+^ permeability (epicardial cell) 4.0 ms^-1^

*v_1_* Maximum RyR channel Ca^2+^ permeability (endocardial cell) 2.9 ms^-1^

*v_2_* Ca^2+^ leak rate constant from the NSR 1.74 × 10^-5^ ms^-1^

*v_3_* SR Ca^2+^-ATPase maximum pump rate 0.315 μM ms^-1^

*K_m,up_* Half-saturation constant for SR Ca^2+^-ATPase pump 0.5 μM

*τ_tr_* Time constant for transfer from NSR to JSR 20.0 ms

*τ_xfer_* Time constant for transfer from subspace to myoplasm 8.0 ms

*k_a_^+^* RyR *P_C1_ − P_O1_* rate constant 0.006075 μM^-4^ ms^-1^

*k_a_^-^* RyR *P_O1_ − P_C1_* rate constant 0.07125 ms^-1^

*k_b_^+^* RyR *P_O1_ − P_O2_* rate constant 0.00405 μM^-3^ ms^-1^

*k_b_^-^* RyR *P_O2_ − P_O1_* rate constant 0.965 ms^-1^

*k_c_^+^* RyR *P_O1_ − P_C2_* rate constant 0.009 ms^-1^

*k_c_^-^* RyR *P_C2_ − P_O1_* rate constant 0.0008 ms^-1^

*n*  RyR Ca^2+^ cooperativity parameter *P_C1_ − P_O1_* 4

*m*  RyR Ca^2+^ cooperativity parameter *P_O1_ − P_O2_* 3

**L-type Ca^2+^ Channel Parameters**

Parameter Definition Value

*G_CaL_* Specific maximum conductivity for L-type Ca^2+^ channel 0.2342 mS μF^-1^

*E_Ca,L_* Reversal potential for L-type Ca^2+^ channel 52.0 mV

*K_pc,max_* Maximum time constant for Ca^2+^-induced inactivation 0.11662 ms^-1^

*K_pc,half_* Half-saturation constant for Ca^2+^-induced inactivation 10.0 μM

*K_pcb_* Voltage-insensitive rate constant for inactivation 0.0005 ms^-1^

*I_CaL,max_* Normalization constant for L-type Ca^2+^ current 7.0 pA pF^-1^

**Buffering Parameters**

Parameter Definition Value

[*LTRPN*]*_tot_* Total myoplasmic troponin low-affinity site concentration 70.0 μM

[*HTRPN*]*_tot_* Total myoplasmic troponin high-affinity site concentration 140.0 μM

*k^+^_htrpn_* Ca^2+^ on rate constant for troponin high-affinity sites 0.00237 μM^-1^ ms^-1^

*k^-^_htrpn_* Ca^2+^ off rate constant for troponin high-affinity sites 3.2 × 10^-5^ ms^-1^

*k^+^_ltrpn_* Ca^2+^ on rate constant for troponin low-affinity sites 0.0327 μM^-1^ ms^-1^

*k^-^_htrpn_* Ca^2+^ off rate constant for troponin low-affinity sites 0.0196 ms^-1^

[*CMDN*]*_tot_* Total myoplasmic calmodulin concentration 50.0 μM

[*CSQN*]*_tot_* Total junctional SR calsequestrin concentration 15,000 μM

*K_m_ ^CMDN^* Ca^2+^ half saturation constant for calmodulin 0.238 μM

*K_m_ ^CSQN^* Ca^2+^ half saturation constant for calsequestrin 800.0 μM

**Membrane Current Parameters**

Parameter Definition Value

*C_m_* Specific membrane capacitance 1.0 μF cm^-2^

*F* Faraday’s constant 96.5 C mmol^-1^

*T* Absolute temperature 298 K

*R* Ideal gas constant 8.314 J mol^-1^ K^-1^

*k_NaCa_* Scaling factor of Na^+^-Ca^2+^ exchange (epicardial cell) 234.24 pA pF^-1^

*k_NaCa_* Scaling factor of Na^+^-Ca^2+^ exchange (endocardial cell) 131.76 pA pF^-1^

*K_m,Na_* Na^+^ half saturation constant for Na^+^-Ca^2+^ exchange 87,500 μM

*K_m,Ca_* Ca^2+^ half saturation constant for Na^+^-Ca^2+^ exchange 1,380 μM

*k_sat_* Na^+^-Ca^2+^ exchange saturation factor at very negative potentials 0.1

η Controls voltage dependence of Na^+^-Ca^2+^ exchange 0.35

*I^max^_NaK_* Maximum Na^+^-K^+^ pump current (epicardial cell) 0.704 pA pF^-1^

*I^max^_NaK_* Maximum Na^+^-K^+^ pump current (endocardial cell) 0.6952 pA pF^-1^

*K_m,Nai_* Na^+^ half saturation constant for Na^+^-K^+^ exchange current 21,000 μM

*K_m,Ko_* K^+^ half saturation constant for Na^+^-K^+^ exchange current 1,500 μM

*I^max^_p(Ca)_* Maximum Ca^2+^ pump current (epicardial cell) 0.085 pA pF^-1^

*I^max^_p(Ca)_* Maximum Ca^2+^ pump current (endocardial cell) 0.0595 pA pF^-1^

*K_m,p(Ca)_* Ca^2+^ half saturation constant for Ca^2+^ pump current 0.5 μM

*G_Cab_* Maximum background Ca^2+^ current conductance (epicardial cell) 0.000033 mS μF^-1^

*G_Cab_* Maximum background Ca^2+^ current conductance (endocardial cell) 0.000017 mS μF^-1^

*G_Na_* Maximum fast Na^+^ current conductance 13.0 mS μF^-1^

*G_Nab_* Maximum background Na^2+^ current conductance 0.0026 mS μF^-1^

*G_Kto,f_* Maximum transient outward K^+^ current conductance (epicardial cell) 0.3846 mS μF^-1^

*G_Kto,f_* Maximum transient outward K^+^ current conductance (endocardial cell) 0.1939 mS μF^-1^

*G_Ks_* Maximum slow delayed rectifier K^+^ current conductance 0.00575 mS μF^-1^

*G_Kto,s_* Maximum transient outward K^+^ current conductance (epicardial cell) 0.0 mS μF^-1^

mS μF^-1^

*G_Kur_* Maximum ultra-rapidly delayed rectifier K^+^ current conductance (epicardial cell) 0.3424

mS μF^-1^

*G_Kur_* Maximum ultra-rapidly delayed rectifier K^+^ current conductance (endocardial cell) 0.1405

*G_Kss_* Maximum non-inactivating steady-state K^+^ current conductance (apex) 0.0611 mS μF^-1^

*G_Kr_* Maximum rapid delayed rectifier K^+^ current conductance 0.078 mS μF^-1^

*k_f_* Rate constant for rapid delayed rectifier K^+^ current 0.023761 ms^-1^

*k_b_* Rate constant for rapid delayed rectifier K^+^ current 0.036778 ms^-1^

*G_Cl,Ca_* Maximum calcium-activated chloride current conductance 10.0 mS μF^-1^

*K_m,Cl_* Half saturation constant for Ca^2+^-activated chloride current 10.0 μM

*E_Cl_* Reversal potential for calcium-activated chloride current −40.0 mV

**Contraction Parameters**

Parameter Definition Value

*SL_0_* Initial sarcomere length 2.1 μm

*k_PN_* Transition rate from permissive to non-permissive state 0.045 ms^-1^

*f_XB_* Basic transition rate from weak to strong crossbridge 0.10 ms^-1^

*g_minxb_* Minimum detachment rate from strong to weak crossbridge 0.14 ms^-1^

**Initial Conditions (epicardial cell)**

Parameter Definition Value

*t* Time 0.0 ms

# *V* Membrane potential −76.3108 mV

[Ca^2+^]*_i_* Myoplasmic Ca^2+^ concentration 0.128703 μM

[Ca^2+^]*_ss_* Subspace SR Ca^2+^ concentration 0.128703 μM

[Ca^2+^]*_JSR_* Junctional SR Ca^2+^ concentration 1125.08 μM

[Ca^2+^]*_NSR_* Network SR Ca^2+^ concentration 1125.08 μM

[*LTRPNCa*] Concentration Ca^2+^ bound low-affinity troponin-binding sites 12.3737 μM

[*HTRPNCa*] Concentration Ca^2+^ bound high-affinity troponin-binding sites 126.707 μM

*O* L-type Ca^2+^ channel conducting state 0.854831×10^-11^

*C_1_* L-type Ca^2+^ channel closed state 0.993178

*C_2_* L-type Ca^2+^ channel closed state 0.680457×10^-2^

*C_3_*  L-type Ca^2+^ channel closed state 0.174826×10^-4^

*C_4_* L-type Ca^2+^ channel closed state 0.199631×10^-7^

*I_1_*  L-type Ca^2+^ channel inactivated state 0.253348×10^-10^

*I_2_*  L-type Ca^2+^ channel inactivated state 0.318339×10^-8^

*I_3_*  L-type Ca^2+^ channel inactivated state 0.943466×10^-8^

*P_C1_* Fraction of RyR channels in state *P_C1_* 0.999714

*P_C2_* Fraction of RyR channels in state *P_C2_* 0.263112×10^-3^

*P_O1_* Fraction of RyR channels in state *P_O1_* 0.233877×10^-4^

*P_O2_* Fraction of RyR channels in state *P_O2_* 0.209256×10^-9^

*C_Na3_*  Closed state of fast Na^+^ channel 0.420023

*C_Na2_*  Closed state of fast Na^+^ channel 0.0267137

*C_Na1_*  Closed state of fast Na^+^ channel 0.723752×10^-3^

*O_Na_*  Open state of fast Na^+^ channel 0.414576×10^-5^

*IF_Na_*  Fast inactivated state of fast Na^+^ channel 0.893687×10^-3^

*I1_Na_*  Slow inactivated state 1 of fast Na^+^ channel 0.124299×10^-4^

*I2_Na_*  Slow inactivated state 2 of fast Na^+^ channel 0.909901×10^-7^

*IC_Na2_*  Close-inactivated state of fast Na^+^ channel 0.0329860

*IC_Na3_*  Close-inactivated state of fast Na^+^ channel 0.518643

[Na^+^]*_i_* Myoplasmic Na^+^ concentration 14,564.6 μM

[K^+^]*_i_* Myoplasmic K^+^ concentration 143,419 μM

*a_to,f_*  Gating variable for transient outward K^+^ current 0.482630×10^-2^

*i_to,f_*  Gating variable for transient outward K^+^ current 0.999945

*n_Ks_*  Gating variable for slow delayed rectifier K^+^ current 0.645626×10^-3^

*a_to,s_*  Gating variable for transient outward K^+^ current 0.921658×10^-3^

*i_to,s_*  Gating variable for transient outward K^+^ current 0.995756

*a_ur_*  Gating variable for ultra-rapidly activating delayed rectifier K^+^ current 0.921658×10^-3^

*i_ur_*  Gating variable for ultra-rapidly activating delayed rectifier K^+^ current 0.995756

*a_Kss_*  Gating variable for non-inactivating steady-state K^+^ current 0.921658×10^-3^

*i_Kss_*  Gating variable for non-inactivating steady-state K^+^ current 1.0

*C_K0_* *m*ERG channel closed state 0.996856

*C_K1_* *m*ERG channel closed state 0.156600×10^-2^

*C_K2_* *m*ERG channel closed state 0.101174×10^-2^

*O_K_* *m*ERG channel open state 0.450855×10^-3^

*I_K_* *m*ERG channel inactivated state 0.115611×10^-3^

*P_RyR_* Ca^2+^ release modulation factor 0.358438×10^-13^

SL Sarcomere length 2.096593 μm

N0 Nonpermissive tropomyosin with 0 crossbridges 0.998770

N1 Nonpermissive tropomyosin with 1 crossbridge 0.367612×10^-4^

P0 Permissive tropomyosin with 0 crossbridges 0.112735×10^-3^

P1 Permissive tropomyosin with 1 crossbridge 0.148856×10^-3^

P2 Permissive tropomyosin with 2 crossbridges 0.408484×10^-3^

P3 Permissive tropomyosin with 3 crossbridges 0.523108×10^-3^
